# First isolation and genetic characterization of a *Toxoplasma gondii* strain from a symptomatic human case of congenital toxoplasmosis in Romania

**DOI:** 10.1051/parasite/2013011

**Published:** 2013-03-29

**Authors:** Carmen Anca Costache, Horaţiu Alexandru Colosi, Ligia Blaga, Adriana Györke, Anamaria Ioana Paştiu, Ioana Alina Colosi, Daniel Ajzenberg

**Affiliations:** 1 Department of Microbiology, “Iuliu Haţieganu” University of Medicine and Pharmacy 6 Louis Pasteur street Cluj-Napoca 400349 Romania; 2 Department of Medical Informatics and Biostatistics, “Iuliu Haţieganu” University of Medicine and Pharmacy 6 Louis Pasteur street Cluj-Napoca 400349 Romania; 3 Department of Neonatology, “Iuliu Haţieganu” University of Medicine and Pharmacy 57 Bulevardul 21 Decembrie 1989 Cluj-Napoca 400124 Romania; 4 Department of Parasitology and Parasitic Diseases, Faculty of Veterinary Medicine, University of Agricultural Sciences and Veterinary Medicine 3–5 Mănăştur street Cluj-Napoca 400372 Romania; 5 Centre National de Référence (CNR) Toxoplasmose/Toxoplasma Biological Resource Center (BRC), Centre Hospitalier-Universitaire Dupuytren Limoges 87042 France; 6 INSERM UMR 1094, Neuroépidémiologie Tropicale, Laboratoire de Parasitologie-Mycologie, Faculté de Médecine, Université de Limoges Limoges 87025 France

**Keywords:** Congenital toxoplasmosis, Genotyping, Eastern Europe

## Abstract

Very limited data exists on the genetic diversity of *Toxoplasma gondii* from Eastern Europe. We present the first Romanian case of symptomatic congenital toxoplasmosis in which the *T. gondii* strain was isolated after inoculation in mice of a cerebrospinal fluid sample from a living neonate. The *T. gondii* strain was genotyped with 15 microsatellite markers distributed on 10 of the 14 chromosomes of *T. gondii*. The strain had a type II genotype.

## Abstract (fr)

Il existe très peu de données sur la diversité génétique de *Toxoplasma gondii* en Europe de l’Est. Nous présentons le premier cas humain de toxoplasmose congénitale en Roumanie dans lequel la souche de *T. gondii* a été isolée chez la souris après inoculation d’un échantillon de liquide céphalo-rachidien d’un nouveau-né vivant. La souche de *T. gondii* a été génotypée avec 15 marqueurs microsatellites localisés sur 10 des 14 chromosomes de *T. gondii*. La souche avait un génotype de type II.

## Introduction

*Toxoplasma gondii* (*T. gondii*) is a highly successful parasite, virtually capable of infecting all species of warm-blooded animals worldwide. Understanding genetic variation of *T. gondii* is critical in reducing the selection possibility of more virulent strains that could produce more severe or even new forms of toxoplasmosis [12].

Isolation and genetic characterization of viable *T. gondii* strains have been described mostly in domestic animals from various geographical areas [6, 7, 10, 17, 20, 22]. The isolation and genetic characterization of clinical isolates of *T. gondii* have been predominantly performed in patients with congenital toxoplasmosis or with severe immunodeficiency conditions, as illustrated by several studies conducted in France [2, 4].

The population structure of this cosmopolitan parasite is complex, with distinct geographic patterns [15]. The highest genetic diversity of *T. gondii* has been described in South America, because a combination of a large gene pool and frequent genetic exchanges has generated a wide variety of different genotypes in this area [14, 18, 21]. In contrast to this high genetic variability, in Western Europe, the population structure of *T. gondii* is markedly clonal, with a huge predominance (>90%) of strains belonging to the type II lineage, both in humans and animals [1, 13]. The type III lineage is far less successful than the type II lineage in Western Europe, but can be observed in some cases [13]. Type I strains and the atypical ones that do not fit into the three major lineages are exceptionally collected in Western Europe [5, 8].

In Eastern Europe, little is known about the genetic diversity of *Toxoplasma gondii*. In this geographic region, nonclonal strains of *T. gondii* have so far been isolated only from animal hosts and they have been exceptionally rare: two isolates from Polish chickens, which interestingly have been found to be identical to a nonclonal sheep isolate from Uruguay in South America [11]. To our knowledge, only 10 strains have been isolated and genotyped so far from human hosts living in Eastern Europe. These strains were collected from cases of human congenital toxoplasmosis, nine in Poland [19] and one in Serbia [9]. All of them were identified as type II genotypes. The genotyping of the nine cases of congenital toxoplasmosis from Poland was based on DNA amplified directly from amniotic fluid or from the cerebrospinal fluid of the infants [19]. Viable *T. gondii* was isolated from cord blood of the foetus in Serbia [9]. In both of these studies [9, 19], genotyping was based on restricted fragment length polymorphism, using five markers (*5′SAG2, 3′SAG2, BTUB, SAG3, GRA6*).

The present study is the first genetic characterization of a *T. gondii* strain isolated in Romania and the first characterization of a *T. gondii* strain from Eastern Europe using 15 microsatellite markers.

## Case report

The case we present is that of a premature (32 weeks of gestational age) female neonate, born in July 2011, in Cluj-Napoca, Romania. The child was born spontaneously, in cranial presentation, with an Apgar score of 9/9. Due to IUGR (Intrauterine Growth Restriction), the newborn had decreased subcutaneous tissue, a fat index of 1.8, a skull perimeter of 31 cm and a body weight of 2,000 g. The anterior fontanelle exhibited interior tension and measured 2/2 cm. Microphthalmia, axial hypotonia, and average respiratory distress were also present at birth. A congenital hydrocephalus had been diagnosed at 26 weeks of gestation. Serological investigation of the mother diagnosed an acute toxoplasmosis in the sixth month of pregnancy (IgG and IgM switched from negative in the second month of pregnancy to positive in the sixth month of pregnancy) followed by treatment with spiramycin (Rovamycin^®^) during the last month of pregnancy. Transfontanellar ultrasonography performed at 4 h after birth showed a dilated lateral ventricle compressing the brain mass, with a biventricular diameter at the level of Monroe’s hole of 33.8 mm. A magnetic resonance imaging (MRI) showed a complex brain malformation with agenesis of the corpus callosum, right frontal schizencephaly, and obstructive hydrocephalus. Examination of the eye revealed congenital acute central chorioretinitis of the right eye and sequelae of anterior and posterior uveitis, retinal detachment, and congenital microphthalmia of the left eye.

## Investigation results

Specific serology against *T. gondii* with an enzyme immunoassay performed at 3 days after birth was positive for IgG (titer >1000 IU/mL, positive if >50 IU/mL; DiaPro, Italy) and for IgA (titer = 3.26 IU/mL, positive if >1 IU/mL; BioRad, France), and equivocal for IgM (index = 0.95, positive if >1, equivocal 0.8–1, negative <0.8; BioRad, France). A Western blot (LD-Bio) assay of the serum performed 3 weeks after birth was positive for IgG and IgM.

**Figure 1.**
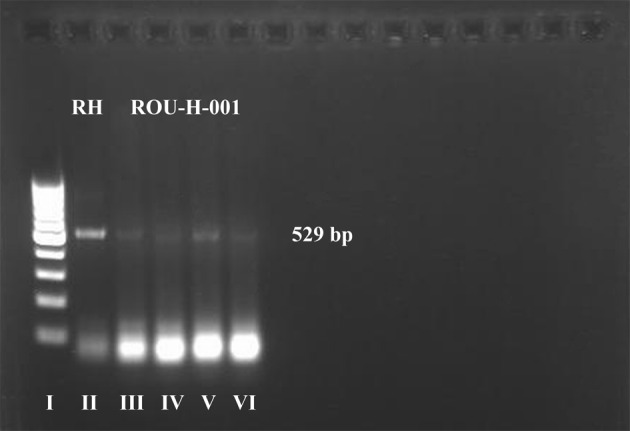
PCR products amplified with specific primer pairs for molecular identification of *T. gondii*, from child’s CSF, migration into agarose gel 1.5%. Lane I – 1 kb DNA ladder; lane II – positive control (RH strain); lanes III–VI – DNA amplified with *T. gondii* specific primers – PCR positive (ROU-H-001 strain).

The cerebrospinal fluid (CSF) was collected 4 days after birth, analyzed by PCR (Figure 1), and bioassayed by mouse inoculation. After DNA extraction of the CSF sample (Qiagen, Germany), a PCR-based assay with primers Tox 4 and Tox 5 detected the 200- to 300-fold repetitive 529 bp DNA fragment of *T. gondii*. Thus, the diagnosis of congenital toxoplasmosis was confirmed and treatment was initiated with spiramycin (Rovamycin^®^, Aventis, France) – 80 mg/kg/day, for 5 weeks after birth, followed by Pyrimethamine (Daraprim^®^, GlaxoSmithKline) – 6 mg/day, in association with folic acid – 1 mg/day and monitoring of hematological parameters.

**Figure 2.**
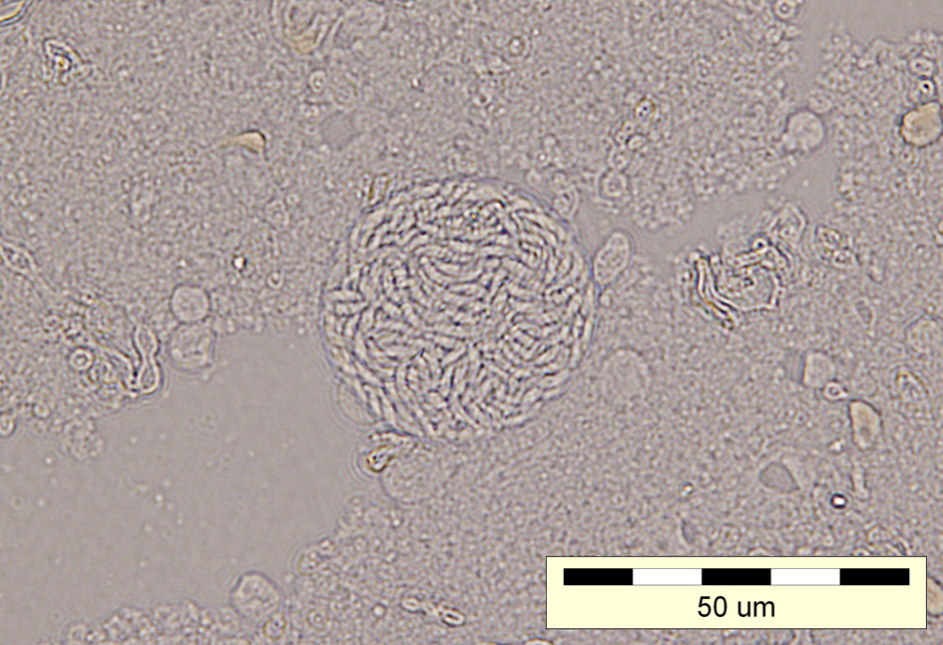
*T. gondii* cyst developing in the brain of an inoculated mouse; microscopic image of a preparation from the occipital lobes, photographed between a cover slide and a glass slide, 4 weeks after inoculation.

The CSF sample was bioassayed into three Swiss white mice. The CSF was centrifuged and resuspended in a saline solution containing penicillin G and streptomycin. This homogenate was inoculated i.p. (0.5 mL/mouse) into three mice. Four weeks post-inoculation all three mice were still asymptomatic, however, the microscopic examination of their brain tissue revealed the presence of *T. gondii* cysts (Figure 2).

The *T. gondii* strain was designated as ROU-H-001 and banked at the *Toxoplasma* Biological Resource Center under the Accession Number TgH113001. It was submitted to DNA extraction (Qiagen, Germany) for a genotyping analysis using 15 microsatellite markers distributed on 10 of 14 chromosomes of *T. gondii*, as described elsewhere [3]. Briefly, for each primer pair, the forward one was 5′-end labeled with fluorescein to allow sizing of PCR products electrophoresed in an automatic sequencer. PCR was carried out in a 25-μL reaction mixture consisting of 12.5 μL of 2X QIAGEN Multiplex PCR Master Mix (Qiagen, France), 5 pmol of each primer, and 5 μL of DNA. Cycling conditions were 15 min at 95 °C; 30 s at 94 °C, 3 min at 61 °C, 30 s at 72 °C (35 cycles), and 30 min at 60 °C. PCR products were diluted 1:10 with deionized formamide. One microliter of each diluted PCR product was mixed with 0.5 μL of a dye-labeled size standard (ROX 500, Applied Biosystems) and 23.5 μL of deionized formamide (Applied Biosystems). This mixture was denatured at 95 °C for 5 min and then electrophoresed using an automatic sequencer (ABI PRISM 3130xl, Applied Biosystems). The sizes of the alleles in bp were estimated using GeneMapper analysis software (version 4.0, Applied Biosystems).

The analyzed *T. gondii* strain had a type II genotype (Table 1).

**Table 1.** Genotyping results of *T. gondii* DNA with 15 microsatellite markers from 5 reference strains collected in America and France, compared to the human strain collected in Romania.

				Microsatellite markers														
Type	Isolate^1^	Origin	Host	*TUB2*	*W35*	*TgM-A*	*B18*	*B17*	*M33*	*IV.1*	*XI.1*	*M48*	*M102*	*N60*	*N82*	*AA*	*N61*	*N83*
I	CT1	USA	Cow	291	248	209	160	342	169	274	358	209	168	145	119	265	87	306
II	TgH32006	France	Human	289	242	207	158	336	169	274	356	215	174	142	111	281	91	310
II	TgA32132	France	Sheep	289	242	207	158	336	169	274	356	221	174	138	111	277	91	312
III	NED	France	Human	289	242	205	160	336	165	278	356	209	190	147	111	267	91	312
Atypical	TgCatBr5	Brazil	Cat	291	242	205	160	362	165	278	356	237	174	140	111	265	89	314
II	ROU-H-001	Romania	Human	289	242	207	158	336	169	274	356	231	176	138	109	273	93	312

1 TgA32132 is also known as FR-OVI-ARI061 strain. All reference strains are available at the *Toxoplasma* Biological Resource Center (http://www.toxocrb.com).

## Discussion

A preliminary genetic comparison of this human *T. gondii* strain with three animal strains collected from the same area of Romania revealed that even though the animal strains were also of type II, they were not identical, exhibiting minor genetic differences at the most polymorphic microsatellite markers. A complete genetic analysis of these animal strains will be published in the near future. Neither the human strain described in this paper nor the three animal strains isolated from the same area of Romania were found to be identical to other type II strains collected in France and genotyped with the same 15 microsatellite markers so far.

## Conclusions

Although genetic data available for *T. gondii* strains from Romania is currently very limited, it would appear that the genetic diversity of *T. gondii* in Eastern Europe may be close to that observed in Western Europe, with a predominance of type II strains in animals and humans, even though minor genetic variations have been detected with microsatellite markers. The question that could be addressed is whether atypical genotypes divergent from the clonal lineage II are more prevalent in Eastern Europe than in Western Europe. This issue is of paramount importance because there is a growing body of data suggesting that atypical strains are more pathogenic in congenital toxoplasmosis than type II strains [8, 16]. There is a need of extending our knowledge regarding the genetic variability distribution of this highly successful parasite. Our results pave the way for conducting further studies in order to isolate and characterize more human and animal *T. gondii* strains from Eastern Europe, as well as other underinvestigated regions of Europe.
